# The S∴π hemibond and its competition with the S∴S hemibond in the simplest model system: infrared spectroscopy of the [benzene-(H_2_S)_*n*_]^+^ (*n* = 1–4) radical cation clusters†
†Electronic supplementary information (ESI) available: Energy-optimized structures of [Bz-(H_2_S)_*n*_]^+^ (*n* = 1 and 2) calculated with the 6-311G+(3df,2p) basis set. Energy-optimized structures of [Bz-(H_2_S)_*n*_]^+^ (*n* = 1 and 2) calculated with the aug-cc-pVDZ basis set. Simulated spectra of [Bz-(H_2_S)_1_]^+^ with the aug-cc-pVDZ basis set. Simulated spectra of [Bz-(H_2_S)_2_]^+^ by the M06-2X and M06-L functionals with the 6-311G+(3df,2p) basis set. Simulated spectra of [Bz-(H_2_S)_2_]^+^ with the aug-cc-pVDZ basis set. Free and H-bonded SH bonds of the ion core of [Bz-(H_2_S)_*n*_]^+^ (*n* = 2–4). Energy-optimized structures of [Bz-(H_2_S)_*n*_]^+^ (*n* = 3 and 4) calculated at B3-LYP-D3/6-311G+(3df,2p). Simulated spectra of [Bz-(H_2_S)_3_]^+^. Simulated spectra of [Bz-(H_2_S)_4_]^+^. Relative energies of stable isomers of [Bz-(H_2_S)_2_]^+^ calculated with the aug-cc-pVDZ basis set. See DOI: 10.1039/c9sc02476j


**DOI:** 10.1039/c9sc02476j

**Published:** 2019-06-19

**Authors:** Dandan Wang, Keigo Hattori, Asuka Fujii

**Affiliations:** a Department of Chemistry , Graduate School of Science , Tohoku University , Sendai 980-8578 , Japan . Email: asuka.fujii.c5@tohoku.ac.jp

## Abstract

The S∴π hemibond (two-center three-electron, 2c-3e, bond) is an attractive interaction between a sulfur atom and π electrons. The S∴π hemibond is of essential importance in understanding chemistry of sulfur radical cations, and its roles in biochemistry have recently attracted much interest. In the present study, we observe the S∴π hemibond in the simplest model system in the gas phase. Infrared spectroscopy is applied to the [benzene-(H_2_S)_*n*_]^+^ (*n* = 1–4) radical cation clusters. In *n* = 1, the CH stretch and SH stretch bands of the benzene and H_2_S moieties, respectively, are clearly different from those of the neutral molecules but similar to those of the ionic species. These vibrational features show that the positive charge is delocalized over the cluster due to the S∴π hemibond formation. In *n* = 2–4, the S∴S hemibond and S–π–S multicenter hemibond (three-center five-electron, 3c-5e, bond) can compete with the S∴π hemibond. The observed vibrational features clearly indicate that the S∴S hemibond formation is superior to the S∴π hemibond and S–π–S multicenter hemibond. Calculations of several dispersion-corrected density functionals are compared with the observations. While all the tested functionals qualitatively catch the feature of the S∴π hemibond, the energy order among the isomers of the different hemibond motifs strongly depends on the functionals. These results demonstrate that the [benzene-(H_2_S)_*n*_]^+^ clusters can be a benchmark of density functionals to evaluate the sulfur hemibonds.

## Abstract 2

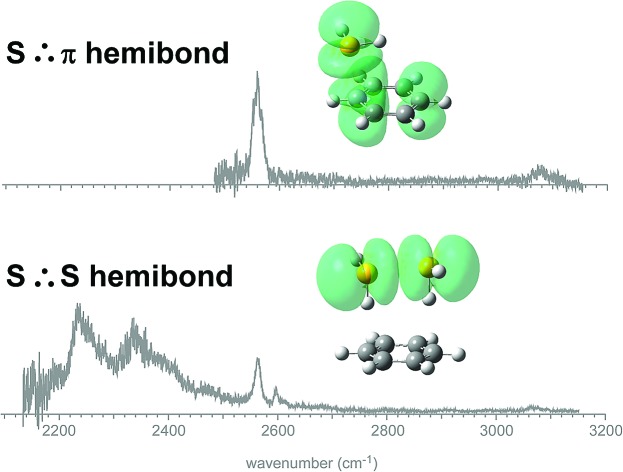
IR spectroscopy of [benzene-(H_2_S)_*n*_]^+^ (*n* = 1–4) elucidates the change of the positive charge accommodation motif from the S∴π hemibond to the S∴S hemibond.

## Introduction

The S∴π hemibond (S–π interaction or two-center three-electron, 2c-3e, bond) is an attractive interaction between a singly occupied lone pair orbital of sulfur and a doubly occupied π orbital, or *vice versa* (here, we should note that the whole benzene molecule with the delocalized π-orbital is regarded as one “center” in the 2c-3e bond while another center is the sulfur atom). The S∴π hemibond has attracted great interest because it plays crucial roles in chemistry and biochemistry of sulfur radical cations.^1^–^10^ For example, it has been pointed out that increased strength of interactions of thioethers and arenes, *e.g.*, methionine–phenyl, facilitates oxidation through the stabilization by the S∴π hemibond formation.^2^–^4^ The S∴π hemibond between a sulfur-containing residue and a phenyl ring in oxidized protein has been supposed to serve as a potent relay station in multistep electron hopping processes.^5^–^7^ Despite the increasing interest to the S∴π hemibond, especially in biochemistry, its spectroscopic evidence has been rather scarce. Experimental confirmation of the S∴π hemibond has been pioneered by Werst with EPR spectroscopy.^8^ Broad electronic transitions due to the excitation to the hole in the anti-bonding orbital have also been used to confirm the formation of the S∴π hemibond in the condensed phase,^9^,^10^ as well as many reports on S∴S hemibonds.^11^ Bally, Glass, and coworkers have performed photoelectron spectroscopy of thioether compounds carefully designed to explore the S∴π hemibond, and the comparison of the spectra of the compounds with and without the phenyl group, as well as with and without the sulfide group, has clearly evidenced the orbital interaction between the sulfur and phenyl ring.^9^

For characterization of an intermolecular interaction, gas phase spectroscopy of its model clusters can provide the most reliable data, which can be also compared directly with high precision quantum chemical computations.^12^ With the proper choice of the combination of molecules in the cluster, factors other than the intermolecular interaction of interest can be excluded. Moreover, competition among multiple intermolecular interactions can be also studied by the choice of the component of the cluster. While for the S∴S hemibond and π∴π hemibond (ordinarily called “charge resonance”), detailed spectroscopic studies on their simple model cluster systems have been reported,^13^–^18^ no such a study has been performed for the S∴π hemibond. Then, in the present work, the radical cation clusters of benzene (Bz) and hydrogen sulfide, [Bz-(H_2_S)_*n*_]^+^, (*n* = 1–4), are studied by infrared (IR) spectroscopy in the SH and CH stretch regions. Since IR spectroscopy is sensitive to molecular structures and intermolecular interactions, IR spectroscopy of S∴π hemibonded systems would provide us rich information on the nature of the S∴π hemibond, which is complementary to the previous electronic and photoelectron studies.^9^,^10^ The *n* = 1 cluster can be the simplest prototype of the S∴π hemibond in the gas phase. The observed vibrational features clearly reveal the S∴π hemibond formation in the radical cation cluster. In the *n* = 2–4 clusters, other hemibond motifs, the S∴S hemibond and S–π–S multicenter hemibond (three-center five-electron, 3c-5e, bond) can compete with the S∴π hemibond. The S∴S hemibond formation in (H_2_S)_*n*_^+^ has been observed by the transient electronic absorption in aqueous solution,^19^ and has recently been confirmed by IR spectroscopy in the gas phase clusters.^13^ Formation of a multicenter hemibond (3c-5e bond) has been reported for rare gas atom clusters, alkaline earth atom clusters, and boryl radicals.^20^–^25^ However, such charge delocalization over two molecules has never been discussed on the S∴π hemibonded system, to our best knowledge. The observed IR spectra of the *n* = 2–4 clusters show that S∴S hemibond formation among H_2_S molecules is superior to the S∴π hemibond and S–π–S multicenter hemibond. The observed spectra are also compared with the spectral simulations by density functional theory (DFT) calculations. As has been pointed out, the validity of DFT calculations of hemibonds strongly depends on functionals.^26^–^30^ Several dispersion-corrected functionals are tested, and it is demonstrated that the [Bz-(H_2_S)_*n*_]^+^ clusters can be a benchmark to evaluate the performance of functionals on the simulation of the hemibond motifs.

## Experimental

Two different preparation methods were applied for [Bz-(H_2_S)_1_]^+^ to test the existence of its stable isomers. One method is resonance-enhanced multiphoton ionization (REMPI) of the neutral Bz-(H_2_S)_1_ cluster under the collision free condition. The gaseous mixture of He/H_2_S/Bz was expanded to a vacuum chamber, and the resultant supersonic jet was skimmed to form a molecular beam. The [Bz-(H_2_S)_1_]^+^ radical cation was prepared by one-color REMPI of neutral Bz-(H_2_S)_1_*via* its S_1_–S_0_ 610 band.^31^ The produced ion structure can be restricted by the vertical ionization from the structure of the neutral cluster. The microwave and IR spectroscopies of jet-cooled Bz-(H_2_S)_1_ have revealed that H_2_S locates on the C_6_ axis of the aromatic ring in S_0_,^31^,^32^ and this structure of neutral cluster might be advantageous to preferentially form the S∴π hemibonded structure of the cation, in which the H_2_S molecule should locate on the phenyl ring. The produced ions were detected by a time-of-flight mass spectrometer. An IR spectrum of the [Bz-(H_2_S)_1_]^+^ radical cation was measured by IR dissociation spectroscopy. The IR light pulse was introduced 50 ns after the ionization light pulse, and the IR light frequency was scanned. The depletion of the [Bz-(H_2_S)_1_]^+^ signal due to the vibrational predissociation was detected as a measure of the IR absorption.

Another method is ionization of bare Bz molecules in the collisional region of the jet expansion. Bare Bz cations were first produced by REMPI of bare neutral Bz, and the [Bz-(H_2_S)_1_]^+^ cluster ion was generated by following collisions with H_2_S in the supersonic jet expansion. In this “pick-up” method, the most stable structure tends to be produced.^33^ The produced ions were introduced into a tandem type quadrupole mass spectrometer.^34^ The cluster ion mass-selected by the first mass spectrometer was irradiated by the IR light in the octopole ion guide. The fragment ion was produced by predissociation following the IR absorption, and was detected by the second mass spectrometer. By measuring the fragment ion intensity while the IR frequency was scanned, an IR spectrum of the parent ion was obtained. The fragment detection can be free from the background signal. Therefore, the quality of observed spectra is less sensitive to the fluctuation of the parent ion intensity in the fragment ion detection than in the depletion detection of the parent ion. The *n* = 2–4 cluster ions were also produced by the pick-up method and their IR spectra were measured by photodissociation spectroscopy using the tandem quadrupole mass spectrometer. In all the IR spectral measurements of *n* = 1–4 by using the tandem quadrupole mass spectrometer, the [Bz-(H_2_S)_*n*–1_]^+^ fragment cation was monitored. No signal was detected in the (H_2_S)_*n*_^+^ fragment channel.

In our previous studies on the sulfur-containing charged clusters, (H_2_S)_*n*_^+^ (*n* = 3–6) and H^+^(H_2_S)_*n*_^+^ (*n* = 3–9),^13^,^35^ we have demonstrated that MP2 calculations show the best performance to reproduce their observed IR spectra. In the present study on [Bz-(H_2_S)_*n*_]^+^, however, we failed in MP2 calculations because of the significant spin contamination. Therefore, we employed DFT to calculate energy-optimized structures and their IR spectra. The four dispersion-corrected functionals, B3LYP-D3, M06-2X, M06-L, and ωB97X-D, were used with the 6-311G+(3df,2p) and aug-cc-pVDZ basis sets. Energy-optimized structure search and harmonic vibrational simulations were performed by the Gaussian 09 and 16 program suites.^36^,^37^

## Results and discussion

**Fig. 1:**
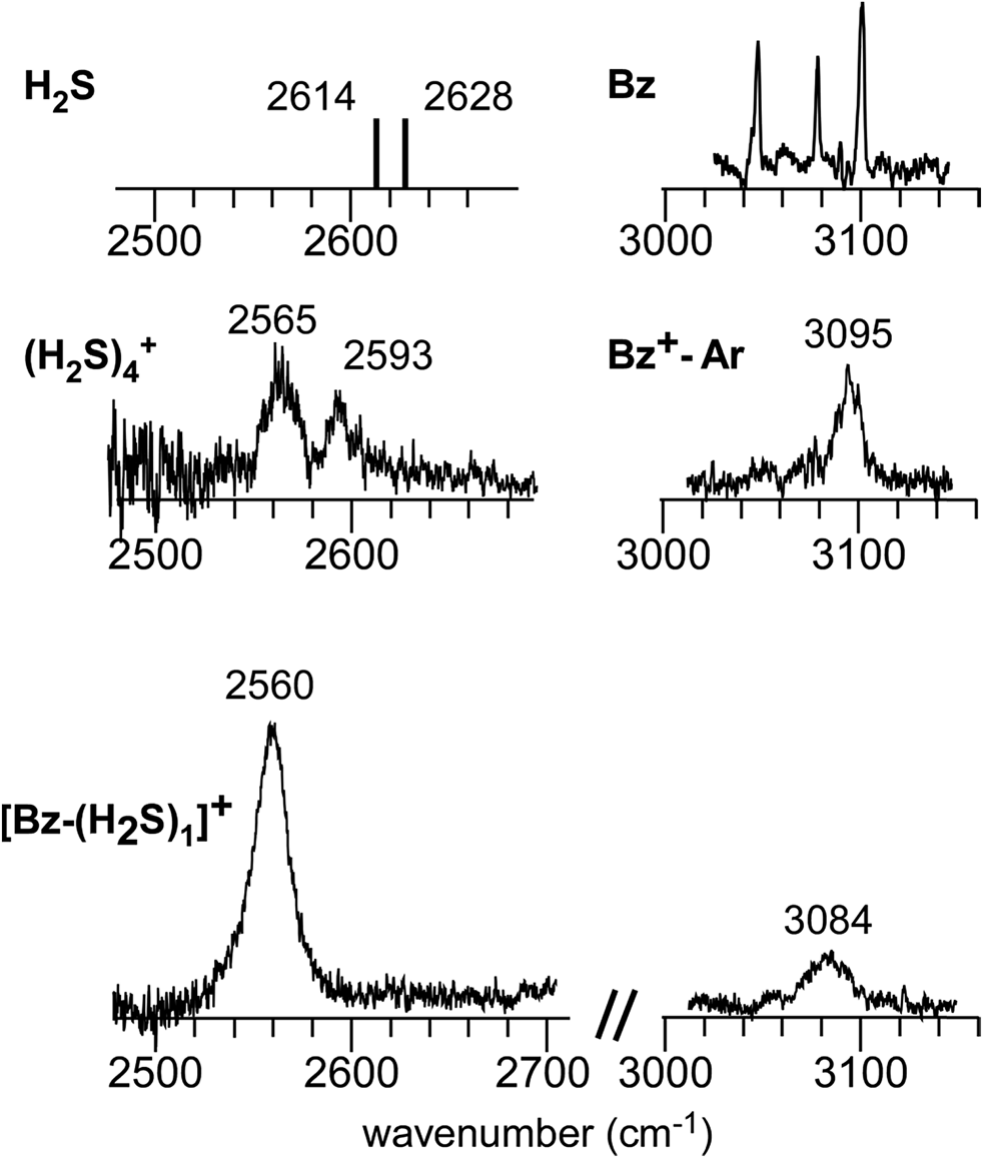
(Bottom) Observed IR spectra of [Bz-(H_2_S)_1_]^+^ in the SH and CH stretch regions. The cluster ion was generated by REMPI of the neutral Bz-(H_2_S)_1_ cluster (see text for details). The depletion spectra are shown inverted in the presentation. (Middle) SH stretch and CH stretch bands of (H_2_S)_4_^+^ and Bz^+^-Ar, respectively. (Top) SH stretch and CH stretch bands of neutral H_2_S and Bz, respectively. The data on H_2_S and (H_2_S)_4_^+^ are taken from ref. 41 and 13, respectively. The relative intensities among these five spectra are arbitrarily scaled.

Fig. 1 shows the CH and SH stretch regions of the [Bz-(H_2_S)_1_]^+^ radical cation prepared through REMPI of neutral Bz-(H_2_S)_1_ in the molecular beam. To qualitatively interpret the spectrum of [Bz-(H_2_S)_1_]^+^, IR spectra of bare neutral Bz^38^ and Bz^+^-Ar^39^,^40^ in the CH stretch region and those of bare neutral H_2_S^41^ and (H_2_S)_4_^+^^13^ in the SH region are also shown in Fig. 1. It has been known that bare Bz in the neutral ground state shows three bands in the CH stretch region because of the Fermi mixing.^38^ In the spectrum of (Bz-H_2_S)^+^, this Fermi triad disappears and only a single weak band is found at 3084 cm^–1^. This feature rather resembles that in Bz^+^-Ar at 3095 cm^–1^, which is supposed to be essentially identical with the bare Bz cation.^39^,^40^ Thus, the CH stretch region suggests that the Bz moiety in [Bz-(H_2_S)_1_]^+^ should be charged, as easily expected by the lower ionization energy of Bz (9.24 eV) than H_2_S (10.46 eV).^42^ In the SH stretch region, a single band is observed at 2560 cm^–1^ and this band is largely red-shifted from those of neutral H_2_S.^41^ This shift cannot be attributed to the π-hydrogen bond formation between H_2_S and Bz. This is because the aromatic ring is positively charged, as shown by its CH stretch band, and the aromatic ring rather repels the proton (hydrogen) of H_2_S.^43^,^44^ When H_2_S directly solvates a charged site, charge transfer occurs more or less, and it lowers the SH stretch frequencies because of the partial reduction of the electron density in the SH bonds. The IR spectra of (H_2_S)_*n*_^+^ (*n* = 3–6) and H^+^(H_2_S)_*n*_^+^ (*n* = 3–9)^13^,^35^ have showed that free SH stretch bands of an essentially neutral H_2_S molecule in the first solvation shell of a charged site are seen only in the region higher than 2585 cm^–1^. The 2593 cm^–1^ band of (H_2_S)_4_^+^ reproduced in Fig. 1 is assigned to the free SH stretch band of such neutral H_2_S molecules in the first solvation shell to the ion core. The observed SH band frequency of [Bz-(H_2_S)_1_]^+^ is too low to be assigned to neutral H_2_S, but it is very close to that of the free SH stretch (2565 cm^–1^) of the ion core moiety in (H_2_S)_4_^+^.^13^ It has been shown that (H_2_S)_4_^+^ has the hemibonded ion core, (H_2_S∴SH_2_)^+^, in which the positive charge is equally shared by the two H_2_S molecules.^13^ Hence, the SH stretch feature suggests that the positive charge in [Bz-(H_2_S)_1_]^+^ is largely shared by the H_2_S moiety, *i.e.*, an S∴π hemibond is formed in the cation. The CH stretch frequency in (Bz)_2_^+^, in which the two Bz molecules are π∴π hemibonded (in the charge resonance state),^15^ has not yet been clearly determined, but it has been estimated to be 3066 cm^–1^ from the dimer ion core component in the IR spectra of (Bz)_*n*_^+^ (*n* ≥ 3).^16^ The observed CH stretch of [Bz-(H_2_S)_1_]^+^ is actually located in between those of bare Bz^+^ and (Bz)_2_^+^, and this is consistent with the S∴π “hetero” hemibond, in which the excess charge cannot be equally shared by the two different molecules.

**Fig. 2:**
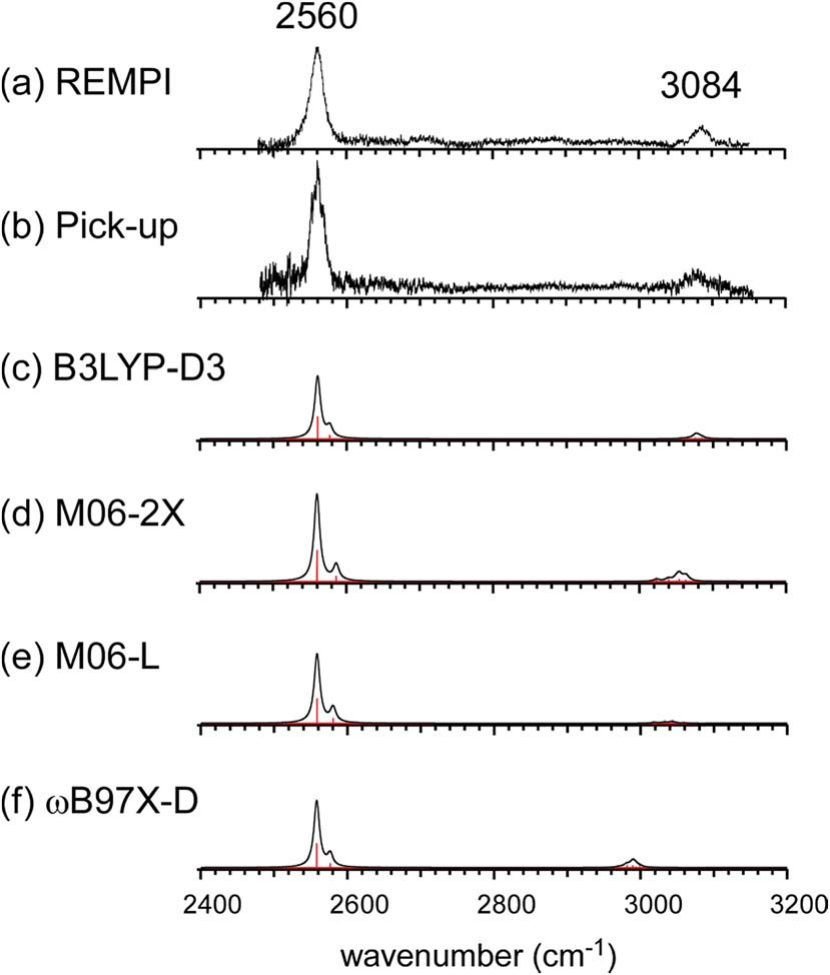
(a and b) Observed IR spectra of [Bz-(H_2_S)_1_]^+^ produced by the REMPI and pick-up type ion sources, respectively. Spectrum (a) is a depletion spectrum shown inverted in the presentation. Spectrum (b) is an enhanced spectrum. (c–f) Simulated IR spectra of [Bz-(H_2_S)_1_]^+^ by B3LYP-D3, M06-2X, M06-L, and ωB97X-D functionals, respectively. The basis set was 6-311G+(3df,2p) for all the computations. The simulated spectra were scaled by the factor of 0.962, 0.948, 0.947, and 0.928, respectively. These scaling factors were determined to fit the observed free SH band at 2560 cm^–1^.

Fig. 2(a) and (b) show the comparison of the observed IR spectra of [Bz-(H_2_S)_1_]^+^ formed by the two different ion sources. Fig. 2(a) is the reproduction of the spectrum of the REMPI produced ion shown in Fig. 1, which is generated by the vertical ionization of the on-top π-hydrogen-bonded neutral cluster. Fig. 2(b) is the spectrum of the ion produced by the pick-up type source. This type of ion source tends to produce more stable ions because of the collisional cooling during the cluster production and no restriction of the initial cluster geometry.^33^ It is clearly seen that the two spectra are essentially identical. This means that there exist no apparent isomers and the S∴π hemibonded structure suggested by the IR spectra would be the most stable structure of [Bz-(H_2_S)_1_]^+^.

This S∴π hemibond formation would be a unique interpretation to reasonably explain the observed IR spectrum of [Bz-(H_2_S)_1_]^+^. To confirm the above qualitative discussion on the observed IR spectrum by the comparison with the related species, dispersion-corrected DFT calculations were performed with four functionals (B3LYP-D3, M06-2X, M06-L, and ωB97X-D) and two basis sets (6-311G+(3df,2p) and aug-cc-pVDZ). Both the basis sets provided similar results for each functionals. Then, in the following, we focus on the results with the 6-311G+(3df,2p) basis set, and those of the aug-cc-pVDZ basis set are summarized in the ESI.†

**Fig. 3:**
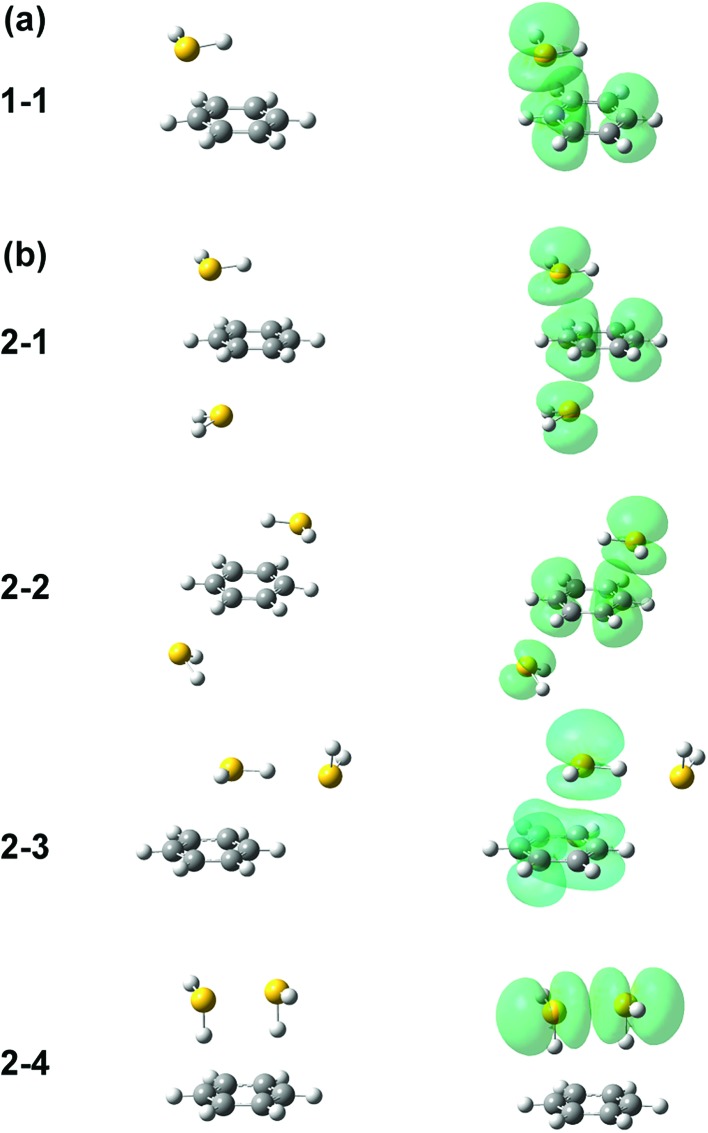
Energy-optimized structures (left column) and spin density plot (right column) of (a) [Bz-(H_2_S)_1_]^+^ and (b) [Bz-(H_2_S)_2_]^+^. All the computations were performed at ωB97X-D/6-311G+(3df,2p). The spin density is plotted at the isovalue density of 0.0004.

Several initial on-top structures as well as in-plane (CH–S hydrogen-bonded type) structures were tried in the energy optimization of [Bz-(H_2_S)_1_]^+^, and all of them finally converged to a unique on-top structure. This is consistent with the missing of apparent isomers in the observed spectra. The stable structure (**1-1**) at the ωB97X-D/6-311G+(3df,2p) level is shown in the left column of Fig. 3(a). The essentially same structure was obtained by the other functionals and basis set, and the results are summarized in ESI.† In this structure, the sulfur atom locates right above a carbon atom and the SH bonds are parallel to the aromatic ring plane. Since the non-bonding orbitals of H_2_S have the 3p character and are almost perpendicular to the SH bonds, the optimized structure suggests large overlap between the non-bonding orbital of H_2_S and the π orbital of Bz. The spin density of this structure is also shown in the right column of Fig. 3(a), and it clearly proves the S∴π hemibond formation, in which the unpaired electron is delocalized over the Bz and H_2_S moieties. The ionization energy of H_2_S is, however, about 1.2 eV higher than Bz, and the charge distribution cannot be equivalent in the two moieties. The natural charges on the H_2_S and Bz moieties are calculated to be 0.438 and 0.562, respectively.

The harmonic vibrational spectra of the S∴π hemibonded structure of [Bz-(H_2_S)_1_]^+^ by the four functionals with the 6-311G+(3df,2p) basis set are shown in Fig. 2(c)–(f). The simulated spectra were scaled to adjust the strongest SH stretch band to the observed band at 2560 cm^–1^, and were also normalized to have the same peak maximum of the SH band. Agreement between the observed and simulated spectra is not perfect; the position and relative peak intensity of the CH stretch band show small differences from the observed ones. However, all the simulated spectra reproduce well the gross features of the SH and CH band positions and their relative intensities of the observed spectra. These simulations strongly support the S∴π hemibond formation in [Bz-(H_2_S)_1_]^+^. Moreover, these simulations demonstrate that all the four functionals are useful to catch the physical essence of the S∴π hemibond.

It is worth to note that the present result on [Bz-(H_2_S)_1_]^+^ is quite different from that of the water analogue cluster, [Bz-(H_2_O)_1_]^+^.^43^,^44^ Also in [Bz-(H_2_O)_1_]^+^, stable on-top structures attributed to the O∴π hemibond have been predicted in the theoretical computations. However, the in-plane structure formed by the CH–O hydrogen bonds is much more stable, and only this structure has been experimentally identified. The finding of the stable in-plane structure in [Bz-(H_2_O)_1_]^+^, which is missing in [Bz-(H_2_S)_1_]^+^, can be attributed the fact that the difference of the ionization energy between Bz and H_2_O (3.4 eV) is much larger than that between Bz and H_2_S (1.2 eV).^42^ Since the exponential dependence of the hemibond strength on the ionization energy difference has been shown,^11^,^45^,^46^ the O∴π hemibond might be much weaker than the S∴π hemibond (therefore, the on-top structure of [Bz-(H_2_O)_1_]^+^ might be essentially regarded as a charge-dipole complex), and the potential minimum in the aromatic ring plane can independently exist. In the in-plane structure of [Bz-(H_2_O)_1_]^+^, the CH stretch band intensity is remarkably enhanced by the CH–O hydrogen bond, and the CH band appears as strong as the OH stretch bands.^44^ This contrasts with the observed intensity distribution of [Bz-(H_2_S)_1_]^+^, in which the SH band is much stronger than the CH band, and also supports the S∴π hemibonded structure of [Bz-(H_2_S)_1_]^+^.

**Fig. 4:**
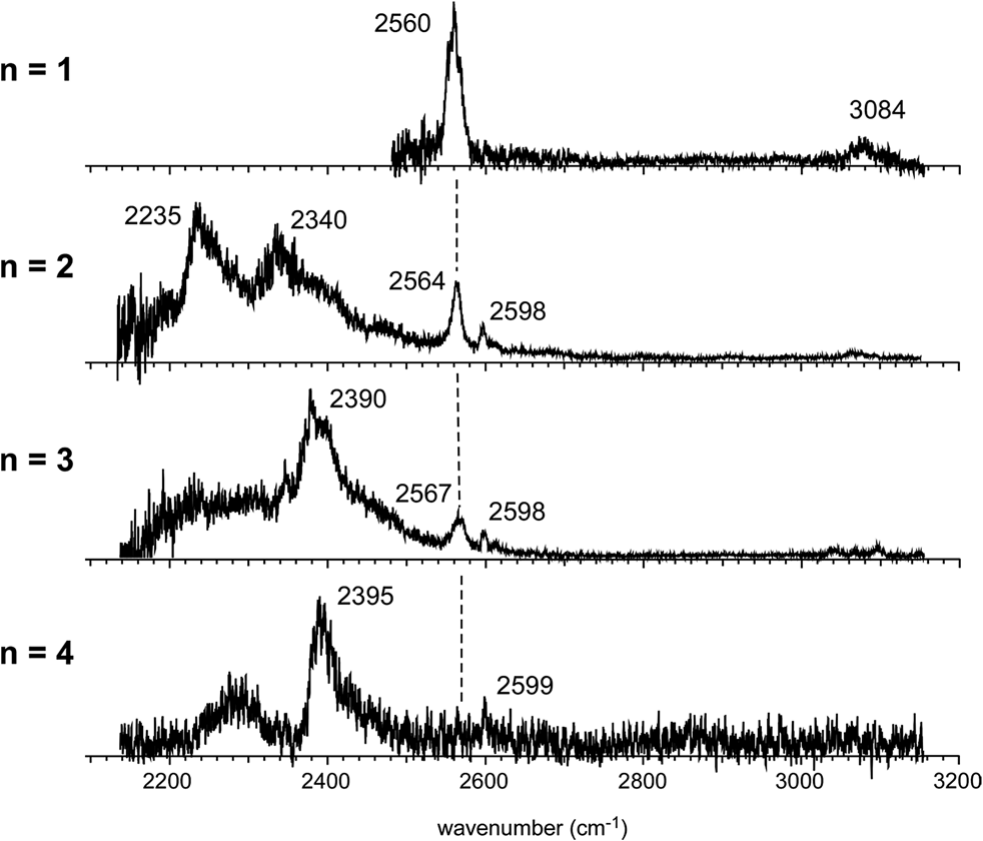
Observed IR spectra of [Bz-(H_2_S)_*n*_]^+^ (*n* = 1–4). All the ions were produced by the pick-up type ion source and the spectra were measured by detecting the enhancement of the [Bz-(H_2_S)_*n*–1_]^+^ fragment ion. The dashed line indicates the free SH stretch band of the ion core.

Fig. 4 shows the IR spectra of [Bz-(H_2_S)_*n*_]^+^ (*n* = 1–4), which are produced by the pick-up type ion source. In the following, the cluster size is simply denoted only with *n*, the number of H_2_S molecules in [Bz-(H_2_S)_*n*_]^+^. In the spectra of the *n* > 1 clusters, several new features are seen, in addition to the free SH of the ion core (∼2560 cm^–1^) and CH stretches of Bz (3000–3100 cm^–1^). By the comparison with the previously reported IR spectra of (H_2_S)_*n*_^+^ and H^+^(H_2_S)_*n*_,^13^,^35^ these new features are unequivocally assigned even without help of quantum chemical computations. The intense and broadened features below 2400 cm^–1^ are attributed to H-bonded SH stretches of the ion core (note that H-bonded SH stretches of neutral H_2_S moieties are generally seen in 2500–2600 cm^–1^).^13^,^47^ The sharp feature (∼2600 cm^–1^) at the high frequency side of the free SH of the ion core is assigned to free SH stretches of the neutral H_2_S moiety.^13^,^35^ In the *n* > 1 clusters, multiple H_2_S molecules enable several different hemibond motifs. The observed IR spectral features provide us rich information on the competition among the hemibond motifs.

Four different types of hemibond motifs are found in the stable structure search of the *n* = 2 cluster at the ωB97X-D/6-311G+(3df,2p) level, and these stable structures and their spin density plots are shown in Fig. 3(b). The essentially same structures were found also with other functionals and basis set, and they are summarized in ESI.† In structure **2-1**, Bz is sandwiched by two H_2_S molecules. The spin density plot shows that the charge is delocalized to all the three molecules, and this means that the S–π–S multicenter hemibond (3c-5e bond) is formed. Structure **2-2** is a variation of the multicenter hemibond; two S∴π hemibonds are formed on the carbon atoms in the diagonal position, and the charge is delocalized to all the molecules. Structure **2-3** holds the single S∴π hemibond. The second H_2_S molecule is essentially neutral and is H-bonded to the hemibonded H_2_S (ion core). In structure **2-4**, the two H_2_S molecules form an S∴S hemibonded ion core, (H_2_S∴SH_2_)^+^, and the ion core is solvated by the neutral Bz molecule. These stable structures demonstrate that three different hemibond motifs, S–π–S, S∴π, and S∴S, can compete in the present system.

The relative energy of each isomer structure is summarized in Table 1. For each functional (row in the table), the energy of the most stable isomer is set to zero. The relative energies of the isomers strongly depend on the functionals, and no common trend among all the functionals can be seen in the table. This means that most of these functionals have serious problems to quantitatively evaluate the hemibond though all of them can qualitatively illustrate the nature of the hemibond motifs.^26^–^30^ In the following, the reliability of these functionals to evaluate the hemibond motifs is examined by comparison with the isomer distribution suggested by the observed IR spectra.

**Table 1:** Relative energies of the stable structures of [Bz-(H_2_S)_2_]^+^ calculated by four different functionals with the 6-311G+(3df,2p) basis set. In the calculations by each functional, the energy of the most stable isomer is set to zero. The zero point energy (ZPE) correction is applied. All units are kJ mol^–1^

	**2-1**	**2-2**	**2-3**	**2-4**
B3LYP-D3	0.0	6.6	5.8	9.5
M06-2X	9.4	(N/A)	7.8	0.0
M06-L	0.0	4.1	13.9	1.5
ωB97X-D	5.3	14.7	0.7	0.0

**Fig. 5:**
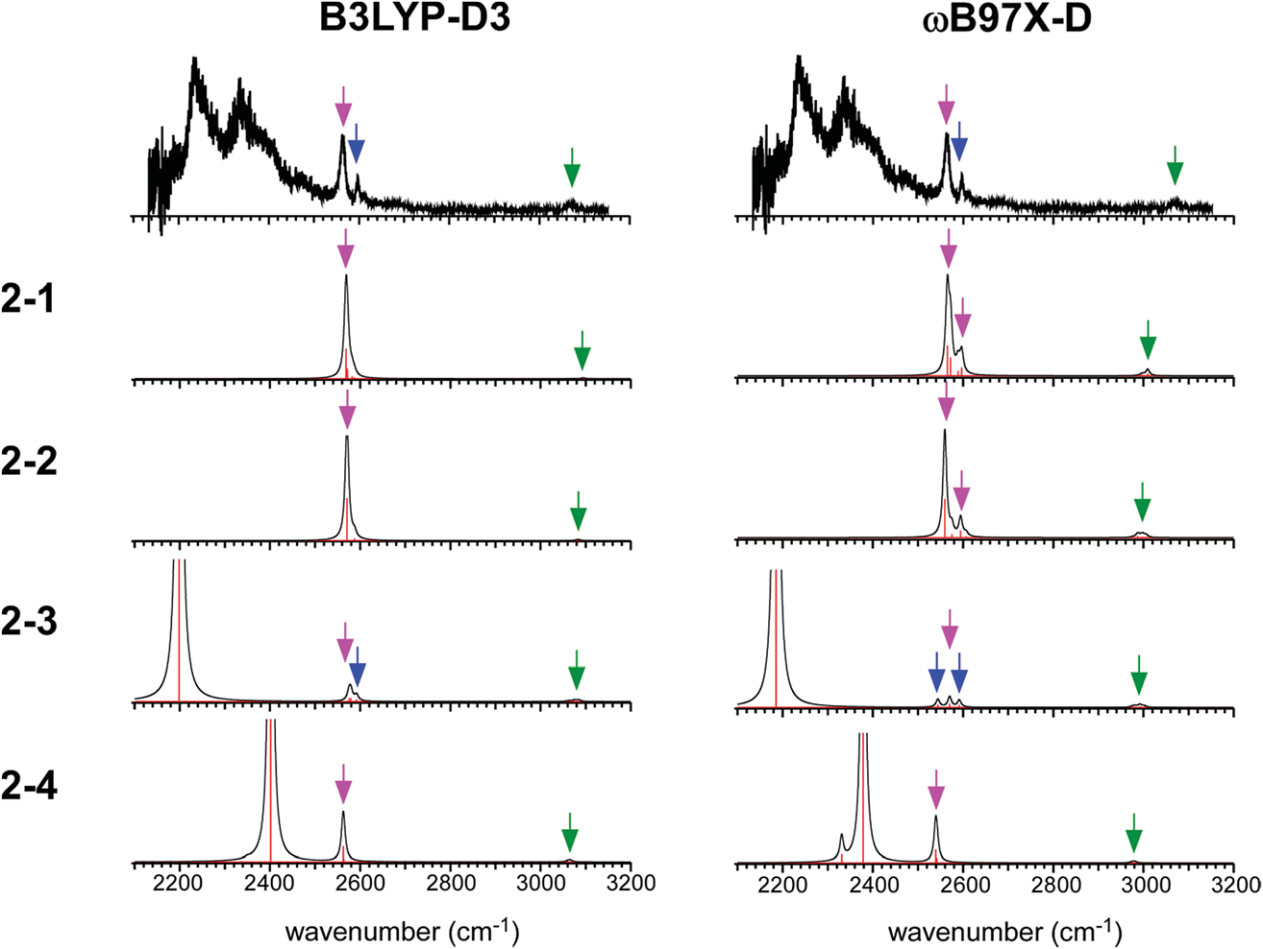
Comparison among the observed IR spectrum of [Bz-(H_2_S)_2_]^+^ and simulated spectra based on the stable isomers. (Left) Simulations by B3LYP-D3/6-311G+(3df,2p). (Right) Simulations by ωB97X-D/6-311G+(3df,2p). The same scaling factors as those in Fig. 2 were applied. The colored arrows present the assignments of the bands; violet: free SH stretch of the ion core, blue: free SH stretch of the neutral H_2_S moiety, and green: CH stretch. Each arrow indicates a peak of an envelope in which contribution of multiple vibrational modes can be involved. Bands without an arrow are attributed to H-bonded SH stretch of the ion core.

The simulated IR spectra of the four isomers of the *n* = 2 cluster at the ωB97X-D/6-311G+(3df,2p) and B3LYP-D3/6-311G+(3df,2p) levels are shown in Fig. 5 with the reproduction of the observed spectrum. The simulations by the M06-2X and M0-6L are summarized in ESI† because their energy evaluations obviously conflict with the observation, as described below. The band assignments are presented by colored arrows; violet: free SH stretch of the ion core, blue: free SH stretch of the neutral H_2_S moiety, and green: CH stretch. Note that each arrow indicates a peak of an envelope in which contribution of multiple vibrational modes can be involved. Bands without an arrow are attributed to H-bonded SH stretch of the ion core, and they appear only below 2400 cm^–1^. The free SH bands of the ion core and neutral H_2_S moiety appear in 2540–2600 cm^–1^, and the band patterns (symmetric/antisymmetric SH stretches or dangling SH stretch) depend on the structures. The splitting between the symmetric and antisymmetric SH stretch bands and their intensity distributions depend also on the functionals.

The observed IR spectra of the *n* = 2 cluster shows two H-bonded SH bands (2235 and 2340 cm^–1^) of the ion core (charged) moiety. To reproduce these two bands in the low frequency region, coexistence of structures **2-3** and **2-4**, each of which shows a single strong H-bonded SH band of the ion core, is clearly requested. In both the M06-2X and M06-L calculations, however, the energy of structure **2-3** relative to structure **2-4** is much higher, and its coexistence is practically excluded. Therefore, the M06-2X and M06-L results conflict with the observation, and these two functionals are omitted in the following discussion. The remaining two functionals, ωB97X-D and B3LYP-D3, provide the totally different energy evaluations of the hemibond motifs. While structures **2-4** and **2-3** are the most stable isomers in ωB97X-D, structure **2-1** is most stable and structure **2-4** is a rather high energy isomer in B3LYP-D3. Structure **2-1** shows only the free SH stretch band of the ion core, which can correspond to the band at 2564 cm^–1^ in the observed spectrum. But this observed band can be also attributed to structures **2-3** and **2-4**. The correct evaluation of the relative intensities of the free SH stretch and H-bonded SH stretch bands is practically difficult from the observed spectrum because of the large intensity difference between these bands. Therefore, we cannot estimate the contribution of structure **2-1** (and/or **2-2**) in the observed spectrum of *n* = 2. The matching of the spectral simulation with the observed spectrum is slightly better in B3LYP-D3 than ωB97X-D. However, the minor difference in the spectral simulations does not affect the assignments of the observed bands, and this cannot be a critical issue in the present case. Further examination of the reliability of the functionals is difficult for *n* = 2. Thus, we examine the spectra of *n* = 3 and 4.

In the observed spectra of *n* = 3 and 4 shown in Fig. 4, the prominent change of the feature is seen in the free SH stretch band of the ion core around 2560 cm^–1^, which is highlighted by the dashed line. This band becomes weaker with increasing cluster size, and finally disappears at *n* = 4, while the free SH stretch band of the neutral moiety is still seen at 2599 cm^–1^. This weakening of the free SH band of the ion core reflects the progress of its solvation (H-bond formation) by H_2_S. The ion core of structure **2-1** (**2-2**) is the whole cluster. Both the two H_2_S molecules are not H-bonded at all, and they have totally 4 free SH bonds. In structure **2-3**, the H_2_S molecule in the ion core (H_2_S bound by the S∴π hemibond) is H-bonded to a neutral H_2_S molecule. Therefore, the ion core has only 1 free SH bond. In structure **2-4**, the ion core is the S∴S hemibonded (H_2_S)_2_^+^ dimer moiety. This ion core is bound to the Bz moiety with the two SH/π H-bonds, and the core has totally 2 free SH bonds. Therefore, each ion core of structures **2-1**, **2-3**, and **2-4** has 4, 1, and 2 free SH bonds, respectively (structures with the labels of the free and H-bonded SH bonds are summarized in Fig. S6 in ESI†). Because the ion core is positively charged, the acidity of the SH bonds of the core is largely enhanced. The free SH bonds in the ion core should be preferentially solvated by H-bond formation with neutral H_2_S in the progress of solvation (one SH bond is solvated by one neutral H_2_S). Therefore, the free SH stretch band of the ion core should disappear at *n* (total number of H_2_S molecules in the cluster) = 6, 3, and 4 for structure **2-1**, **2-3**, and **2-4** type ion cores, respectively, with the completion of the first solvation shell of the ion core. The observed disappearance of the free SH stretch band of the ion core at *n* = 4 is a clear indication of the structure **2-4** type (S∴S hemibonded) ion core in *n* = 4.

**Fig. 6:**
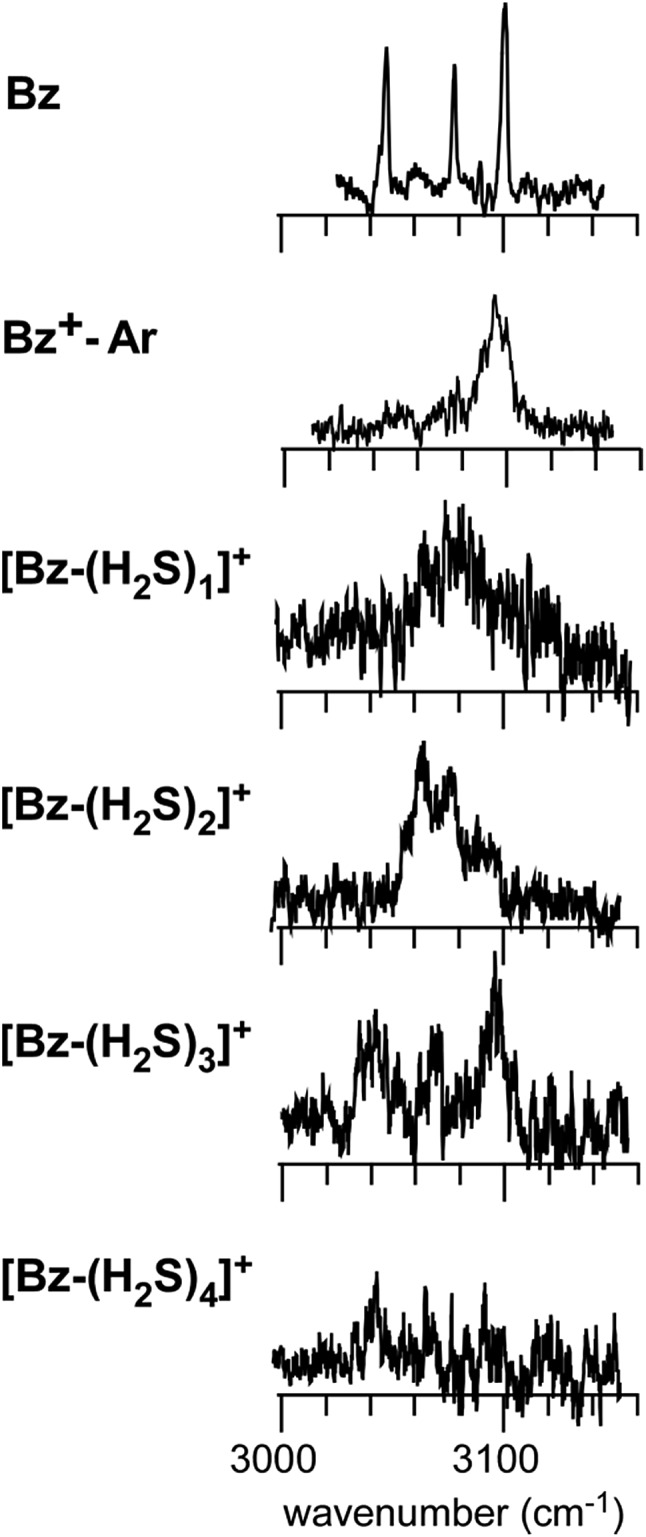
The expanded CH stretch region of the observed spectra of [Bz-(H_2_S)_*n*_]^+^ (*n* = 1–4). Those of neutral Bz and Bz^+^-Ar are also reproduced for comparison.

The preference of the structure **2-4** type ion core is also evidenced by the spectral change in the CH stretch region. The CH stretch region of the observed spectra of the *n* = 1–4 clusters is reproduced in Fig. 6 in the expanded scale. While *n* = 1 shows the single and broadened band, which is quite similar to the CH stretch band of bare Bz^+^ (Bz^+^-Ar),^39^,^40^ the *n* = 3 cluster shows the clear Fermi triad structure, which indicates that the Bz moiety is essentially neutral.^38^ As seen in the spin density plots in Fig. 3(b), among the four ion core structures (stable structures of *n* = 2), only structure **2-4** has the neutral Bz moiety. Therefore the spectra of the CH stretch region demonstrate that the major isomer of *n* = 3 has the structure **2-4** type ion core. Unfortunately, the CH stretch band of the *n* = 4 cluster is hardly seen because of its poor signal to noise ratio.

**Fig. 7:**
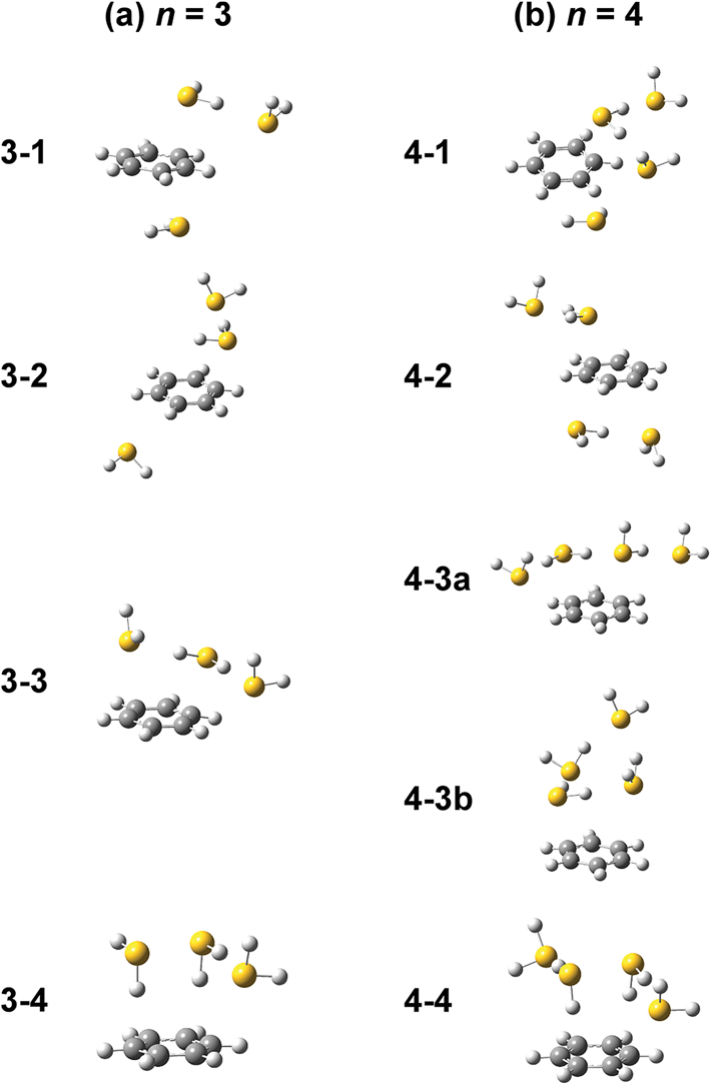
Energy-optimized structures of (a) [Bz-(H_2_S)_3_]^+^ and (b) [Bz-(H_2_S)_4_]^+^ obtained at ωB97X-D/6-311G+(3df,2p).

Computations of stable structures of *n* = 3 and 4 were performed by the ωB97X-D and B3LYP-D3 functionals with the 6-311G+(3df,2p) basis set. As for *n* = 3, the energy optimized structures and their relative energies are summarized in Fig. 7(a) and Table 2, respectively (in Fig. 7(a), only schematic structures obtained at ωB97X-D/6-311G+(3df,2p) are shown. The corresponding stable structures were also obtained at B3LYP-D3/6-311G+(3df,2p) and they were summarized in ESI†). Structures **3-1**, **3-2**, **3-3**, and **3-4** shown in Fig. 7(a) are based on structures **2-1**, **2-2**, **2-3**, and **2-4**, respectively, and one more H_2_S molecule solvates the ion core moiety by an H-bond. In both the computational levels, structure **3-4** of the S∴S hemibonded motif is the most stable isomer. However, other isomers are much higher in energy in ωB97X-D, while structures **3-1**, **3-3**, and **3-4** are almost degenerated in B3LYP-D3. The vibrational simulations based on these structures are summarized in ESI.† Because of the appearance of the free SH stretch band of the ion core (2567 cm^–1^) in the observed spectrum, dominant population of **3-3**, which lacks free SH in the ion core, is easily excluded. As the case of *n* = 2, it is practically difficult to uniquely identify the contribution of isomers by the comparison of the SH stretch region. As shown in Fig. 6, however, the CH stretch region of the observed spectrum clearly demonstrates that the Bz moiety is essentially neutral, and this means exclusive population of structure **3-4**, in which the Bz moiety is not involved in the ion core (here, we should note that the characteristic Fermi triad structure of the CH stretch of neutral Bz cannot be reproduced by the simple harmonic vibrational simulation). Therefore, the ωB97X-D computation well reproduces the observed spectral feature, and the energetics evaluated by B3LYP-D3, which predicts coexistence of structures **3-1**, **3-3**, and **3-4**, is not consistent with the observation.

**Table 2:** Relative energies of the stable structures of [Bz-(H_2_S)_3_]^+^ calculated by two functionals with the 6-311G+(3df,2p) basis set. In the calculations by each functional, the energy of the most stable isomer is set to zero. The zero point energy (ZPE) correction is applied. All units are kJ mol^–1^

	**3-1**	**3-2**	**3-3**	**3-4**
B3LYP-D3	0.68	6.4	0.1	0.0
ωB97X-D	12.8	18.2	4.6	0.0

The energy optimized structures of *n* = 4 and their relative energies are summarized in Fig. 7(b) and Table 3, respectively. Structures **4-1**, **4-2**, and **4-4** are based on structures **3-1** (**2-1**), **3-2** (**2-2**), and **3-4** (**2-4**), respectively. Both structures **4-3a** and **4-3b** are based on **3-3** (**2-3**). In all these structures, the ion core moiety is solvated by one more H_2_S molecule than *n* = 3. In *n* = 4, both the calculation levels show that structure **4-4** which has the S∴S hemibonded ion core is the most stable structure. Other isomers are much higher in energy, and the exclusive population of structure **4-4** is predicted. The vibrational simulations by these structures are also summarized in ESI.† Structures **4-3** and **4-4** have no free SH in the ion core, and well reproduce the missing of the free SH band of the ion core in the observed spectrum. As was already pointed out in the above qualitative discussion, the missing of the free SH band of the ion core occurs at *n* = 4, not at *n* = 3. Moreover, an H-bonded SH stretch band of the neutral moiety is expected in 2500–2600 cm^–1^ region for structure **4-3** (both **a** and **b**), but such a band is missing in the observed spectrum. All these observed features indicate the dominance of structure **4-4** in *n* = 4, and this supports the computational prediction.

**Table 3:** Relative energies of the stable structures of [Bz-(H_2_S)_4_]^+^ calculated by two functionals with the 6-311G+(3df,2p) basis set. In the calculations by each functional, the energy of the most stable isomer is set to zero. The zero point energy (ZPE) correction is applied. All units are kJ mol^–1^

	**4-1**	**4-2**	**4-3a**	**4-3b**	**4-4**
B3LYP-D3	8.7	9.2	11.1	9.1	0.0
ωB97X-D	22.7	21.0	15.6	13.0	0.0

The observed IR spectrum of *n* = 2 shows the coexistence of structures **2-3** (S∴π hemibonded) and **2-4** (S∴S hemibonded), and those of *n* = 3 and 4 demonstrate the clear preference of the S∴S hemibonded type ion core in these sizes. These observations agree well with the energy evaluation of the ωB97X-D functional, but conflict with those of other functionals. Therefore, it is concluded that ωB97X-D is the best performance functional among the present four functionals to evaluate the hemibond motifs. Moreover, the ωB97X-D calculations predict that the S–π–S multicenter hemibond is much less stable than the S∴π and S∴S hemibonds. This less preference of the multicenter hemibond is also supported by the present experimental spectra of *n* = 3 and 4.

## Conclusions

The S∴π hemibond formation and its competition with other sulfur hemibond motifs in the model clusters, [Bz-(H_2_S)_*n*_]^+^ (*n* = 1–4), were studied by IR spectroscopy combined with the DFT computations. The IR spectrum of *n* = 1 clearly demonstrated the formation of the S∴π hemibond in this simplest model system. In *n* = 2–4, the S–π–S multicenter hemibond and S∴S hemibond can compete with the S∴π hemibond. The IR spectrum of *n* = 2 showed the coexistence of the S∴π and S∴S hemibond motifs. The spectral features in *n* = 3 and 4 indicated the S∴S hemibond motif is superior to other hemibond motifs. The relative energy evaluations of these hemibond motifs strongly depend on the DFT functionals, and the ωB97X-D functional showed the best performance to reproduce the observed trend. It should be noted that the charge accommodation motifs in [Bz-(H_2_S)_*n*_]^+^ (*n* = 1–4) can be uniquely assigned by their spectral features essentially without help of theoretical computations. Therefore, these [Bz-(H_2_S)_*n*_]^+^ clusters can be a benchmark system to evaluate performance of DFT functionals to handle sulfur hemibonds. Moreover, they would be highly helpful to explore the nature of the S∴π hemibond by both more extensive experimental and theoretical approaches, and obtained knowledge will be the basis to discuss roles of the S∴π hemibond in biological functions.

## Conflicts of interest

There are no conflicts to declare.

## Supplementary Material

Supplementary information
