# Supplementary material for: The S∴π hemibond and its competition with the S∴S hemibond in the simplest model system: infrared spectroscopy of the [benzene-(H2S)n]+ (n = 1–4) radical cation clusters
Source: Chem Sci. 2019 Jun 19;10(30):7260–8. doi: 10.1039/c9sc02476j (PMC6685351; doi:10.1039/c9sc02476j)
Supplement: Supplementary file 1 [file SC-010-C9SC02476J-s001.pdf]

Electronic Supplementary Material (ESI) for Chemical Science.
This journal is © The Royal Society of Chemistry 2019

Electronic Supplementary Information for

The S∴π hemibond and its competition with the S∴S hemibond in the simplest model system: Infrared spectroscopy of the [benzene-(H_2S)$_n$]$^+$ (n = 1 - 4) radical cation clusters

Department of Chemistry, Graduate School of Science, Tohoku University
Sendai 980-8578, Japan

Dandan Wang, Keigo Hattori, Asuka Fujii*

* Corresponding author: asuka.fujii.c5@tohoku.ac.jp

Contents

Figure S1. Energy-optimized structures of [Bz-(H_2S)$_n$]$^+$ (n = 1 and 2) calculated with the 6-311G+(3df,2p) basis set.

Figure S2. Energy-optimized structures of [Bz-(H_2S)$_n$]$^+$ (n = 1 and 2) calculated with the aug-cc-pVDZ basis set..

Figure S3. Comparison among the observed spectra of [Bz-(H_2S)$_1$]$^+$ and simulated spectra with the aug-cc-pVDZ basis set.

Figure S4. Comparison among the observed spectrum of [Bz-(H_2S)$_2$]$^+$ and simulated spectra by the M06-2X and M06-L functionals with the 6-311G+(3df,2p) basis set.

Figure S5. Comparison among the observed spectrum of [Bz-(H_2S)$_2$]$^+$ and simulated spectra with the aug-cc-pVDZ basis set.

Figure S6. Free and H-bonded SH bonds of the ion core of [Bz-(H_2S)$_n$]$^+$ (n = 2 - 4).

Figure S7. Energy-optimized structures of [Bz-(H_2S)$_n$]$^+$ (n = 3 and 4) calculated at B3-LYP-D3/6-311G+(3df,2p).

Figure S8. Comparison among the observed spectrum of [Bz-(H_2S)$_3$]$^+$ and simulated spectra.

Figure S9. Comparison among the observed spectrum of [Bz-(H_2S)$_4$]$^+$ and simulated spectra.

Table S1. Relative energies of stable isomers of [Bz-(H_2S)$_2$]$^+$ calculated with the aug-cc-pVDZ basis set.

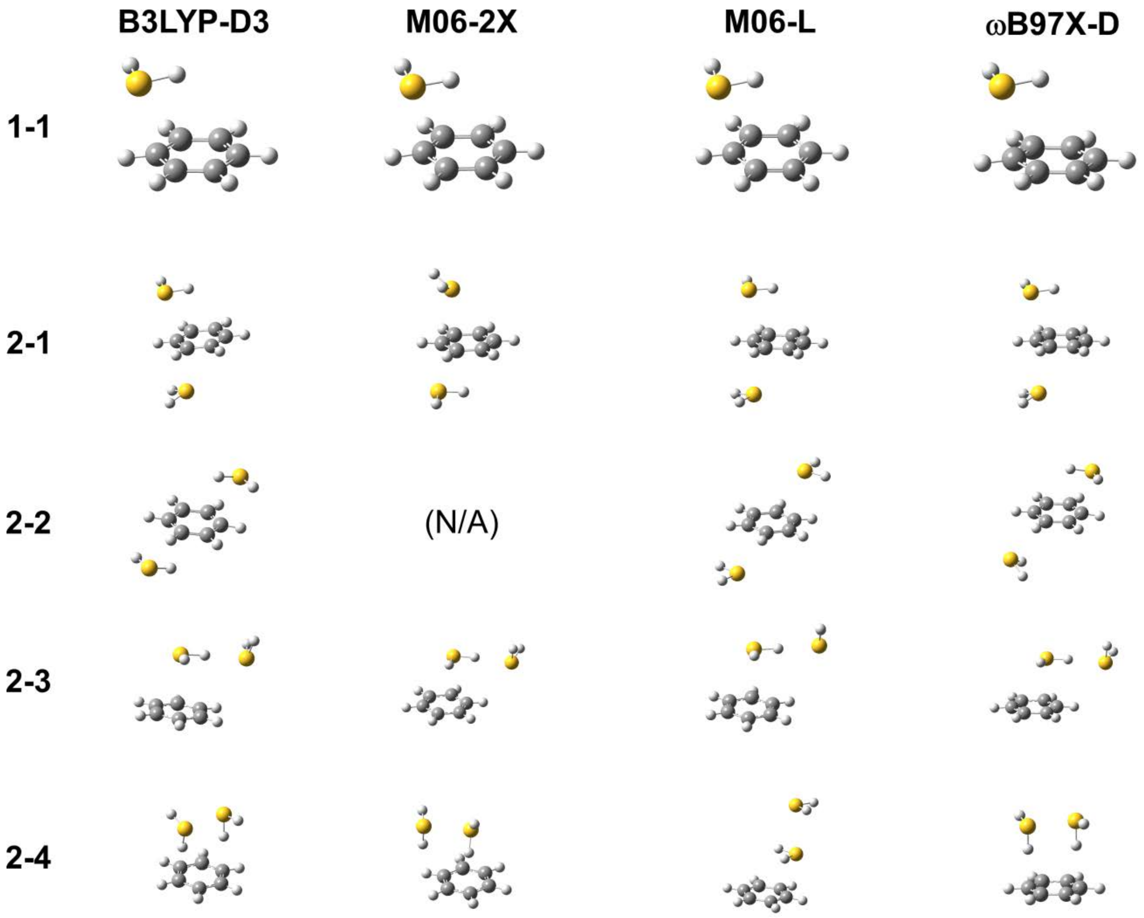

Figure S1. Energy-optimized structures of $[Bz\text{-}(H_2S)_n]^+$ (n = 1 and 2) by the four density functionals. The basis set is 6-311G+(3df,2p).

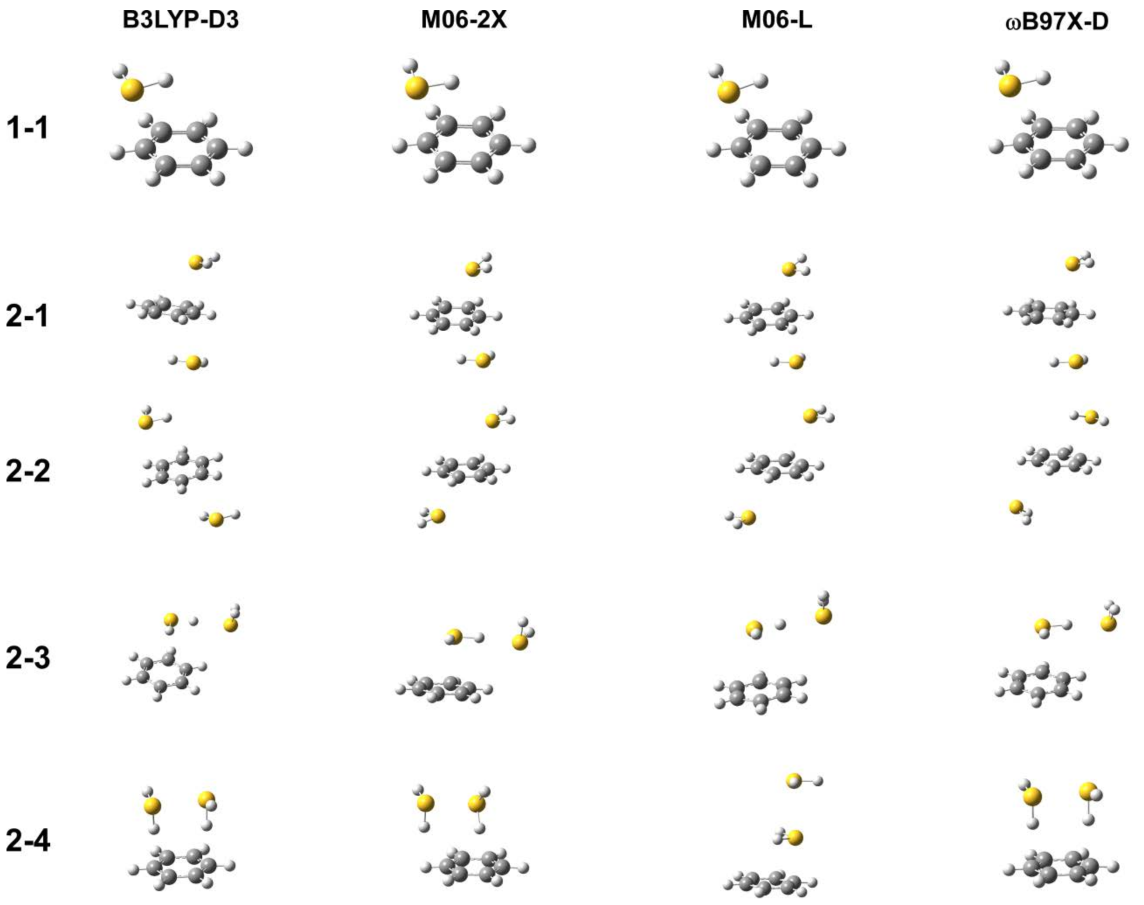

Figure S2. Energy-optimized structures of $[Bz\text{-}(H_2S)_n]^+$ (n = 1 and 2) by the four density functionals. The basis set is aug-cc-pVDZ.

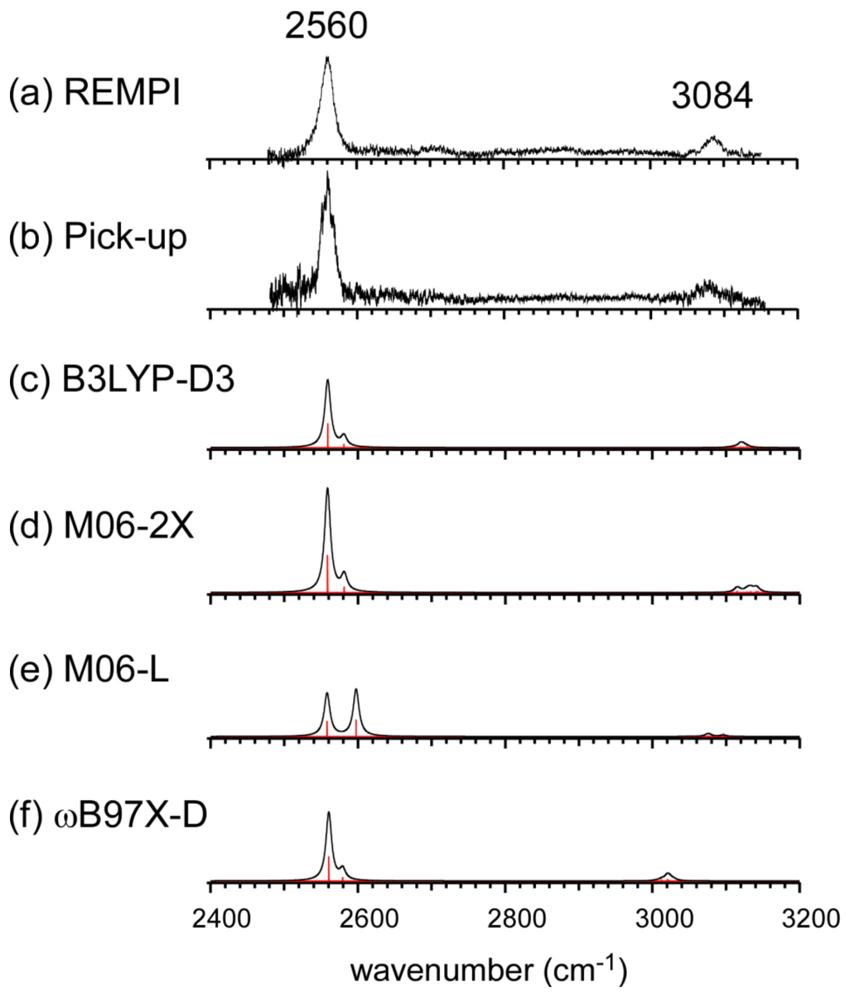

Figure S3. (a), (b) Observed IR spectra of $[Bz\text{-}(H_2S)_1]^+$ produced by the REMPI and pick-up type ion sources, respectively. Spectrum (a) is a depletion spectrum shown inverted in the presentation. Spectrum (b) is an enhanced spectrum. (c)-(f) Simulated IR spectra of $[Bz\text{-}(H_2S)_1]^+$ by the B3LYP-D3, M06-2X, M06-L, and ωB97X-D functionals, respectively. The basis set was aug-cc-pVDZ for all the computations. The simulated spectra were scaled by the factor of 0,972, 0.965, 0.953, and 0.934, respectively. These scaling factors were determined to fit the observed free SH band at 2560 cm^{-1}.

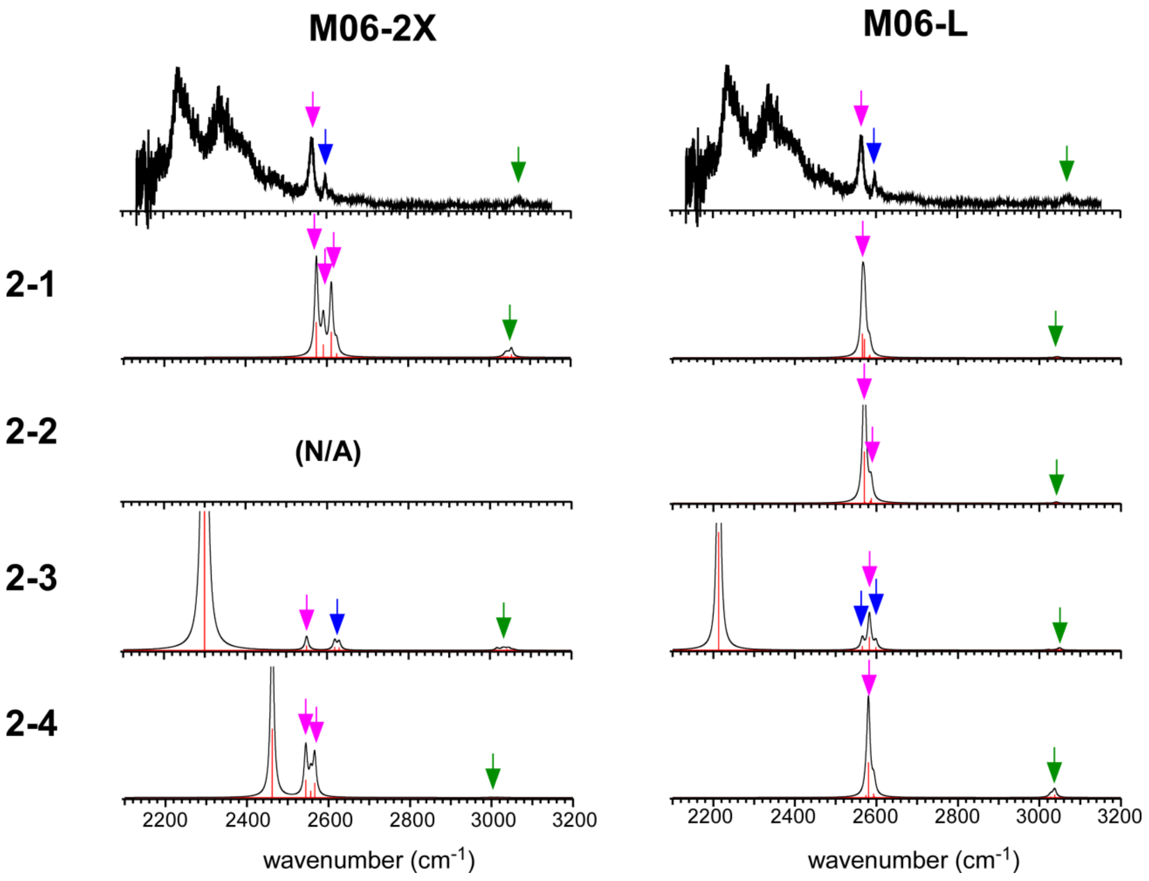

Figure S4. Comparison among the observed IR spectrum of $[Bz\text{-}(H_2S)_2]^+$ and simulated spectra based on the stable isomers. (Left) Simulations by M06-2X/6-311G+(3df, 2p). (Right) Simulations by M06-L/6-311G+(3df, 2p). The same scaling factors as those in Figure 2 in the main text were applied. The colored arrows present the assignments of the bands; violet: free SH stretch of the ion core, blue: free SH stretch of the neutral H_2S moiety, and green: CH stretch. Each arrow indicates a peak of an envelope in which contribution of multiple vibrational modes can be involved. Bands without an arrow are attributed to H-bonded SH stretches of the ion core, and they appear only below 2450 cm^{-1}.

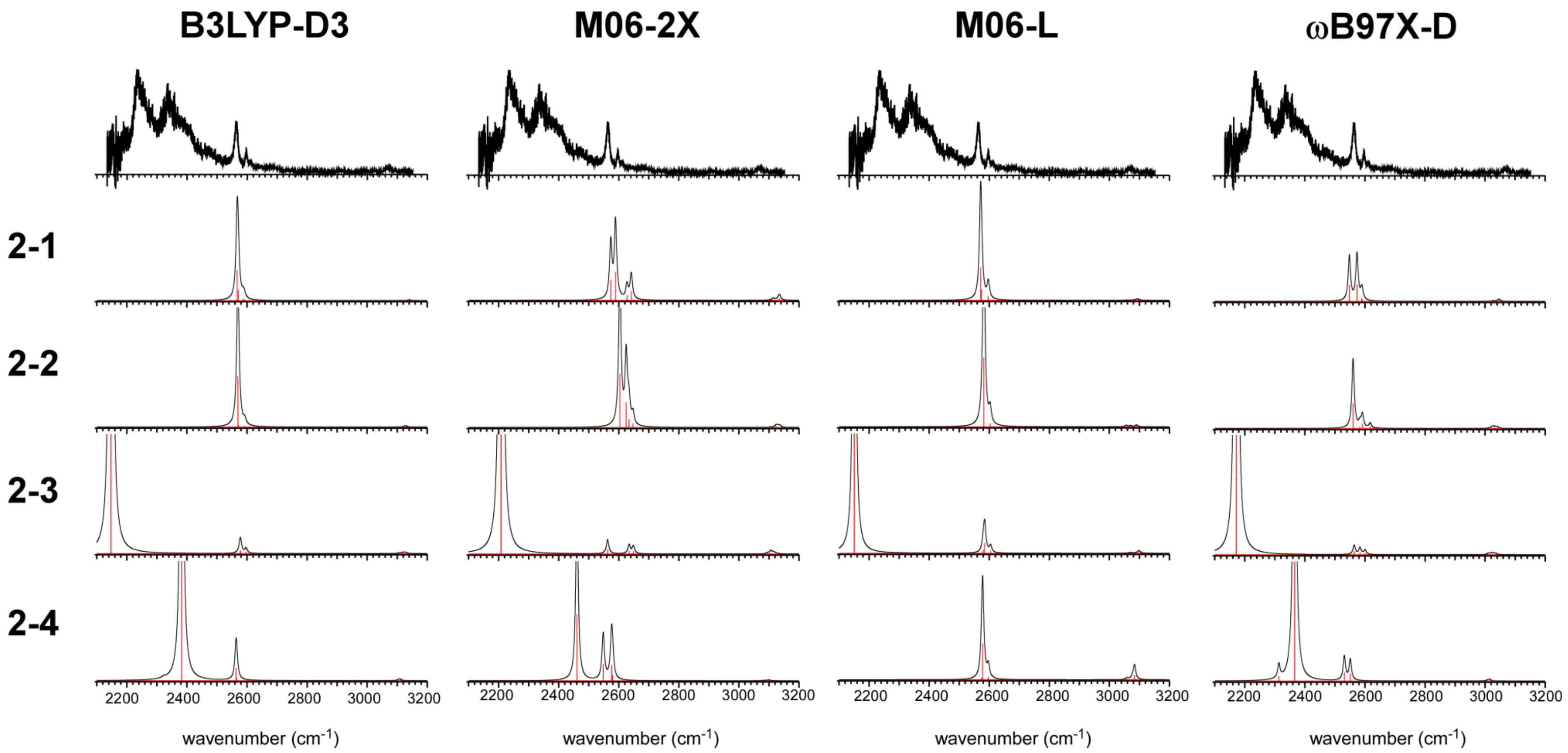

Figure S5. Comparison among the observed IR spectrum of [Bz-(H_2S)$_2$]$^+$ and simulated spectra calculated with the aug-cc-pVDZ basis set. The same scaling factors as those in Figure S3 in ESI were applied.

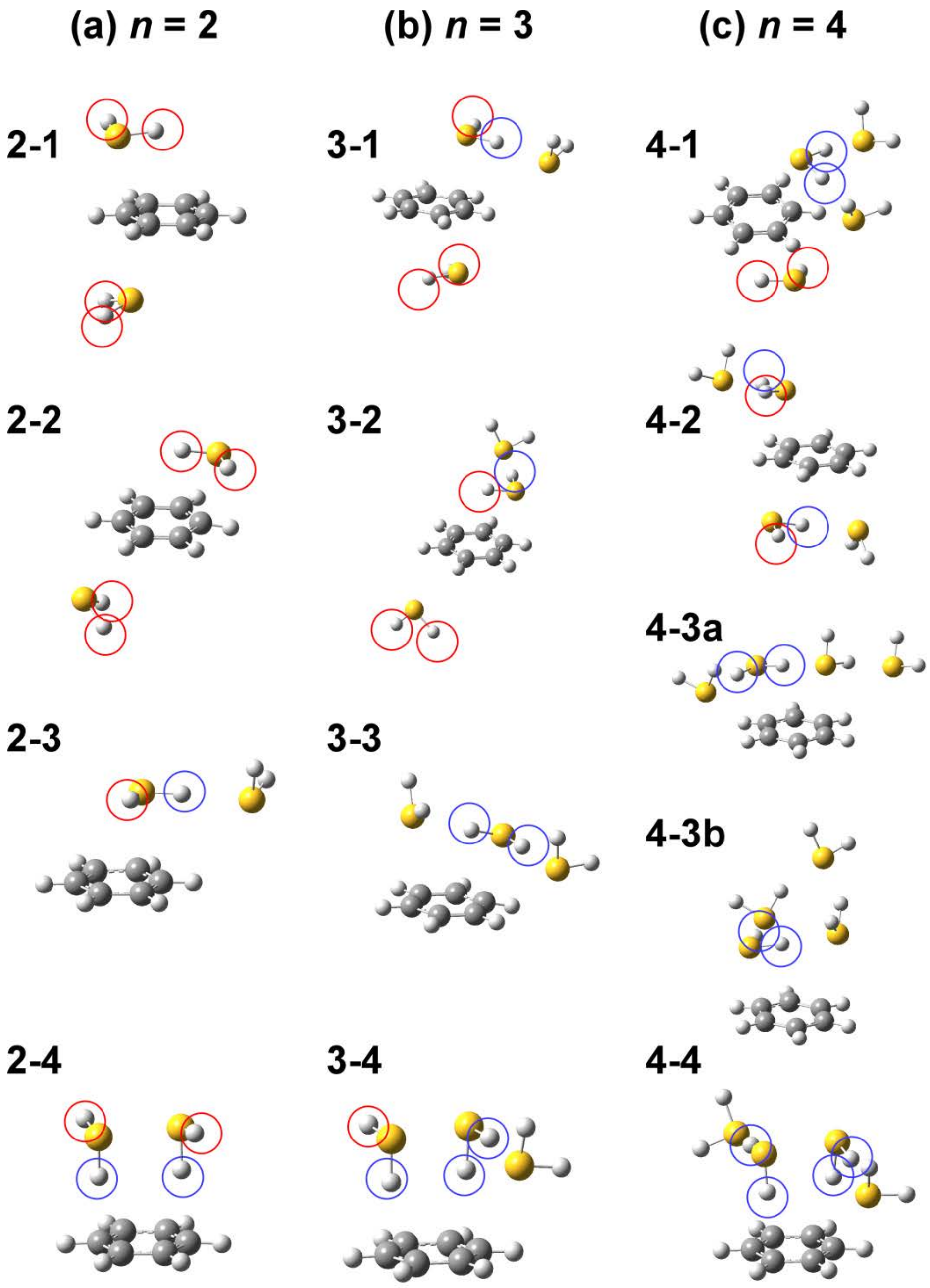

Figure S6. Free and H-bonded SH bonds of the ion core of $[Bz\text{-}(H_2S)_n]^+$ (n = 2 - 4). Red and blue circles indicate free and H-bonded SH of the ion core, respectively. SH bonds without markers are those of neutral solvent molecules. Structures are taken from the energy-optimized structures calculated at ωB97X-D/6-311G+(3df,2p) (shown in Figures 3 and 7 in the main text).

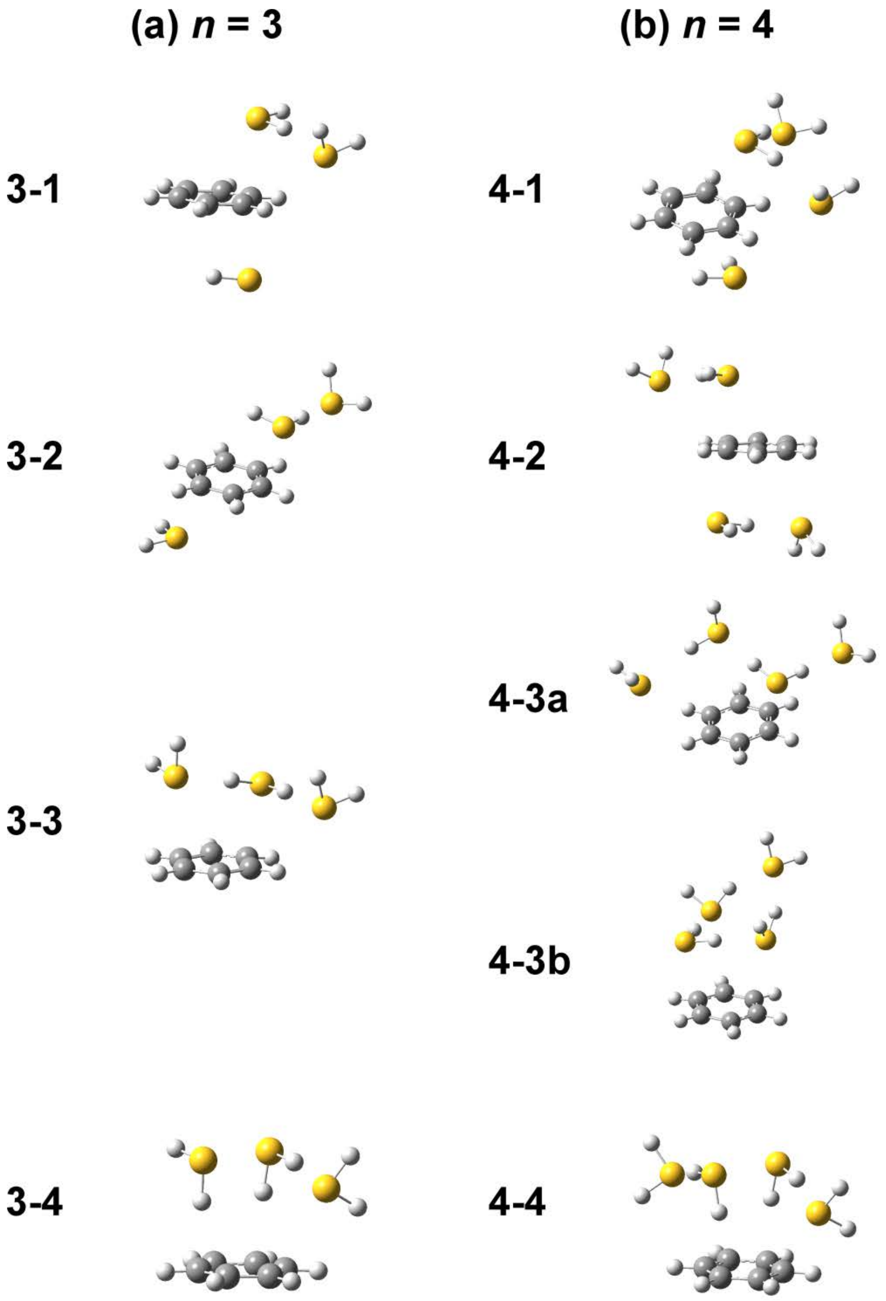

Figure S7. Energy-optimized structures of $[Bz\text{-}(H_2S)_n]^+$ ((a) n = 3 and (b) n = 4) calculated at B3-LYP-D3/6-311G+(3df,2p).

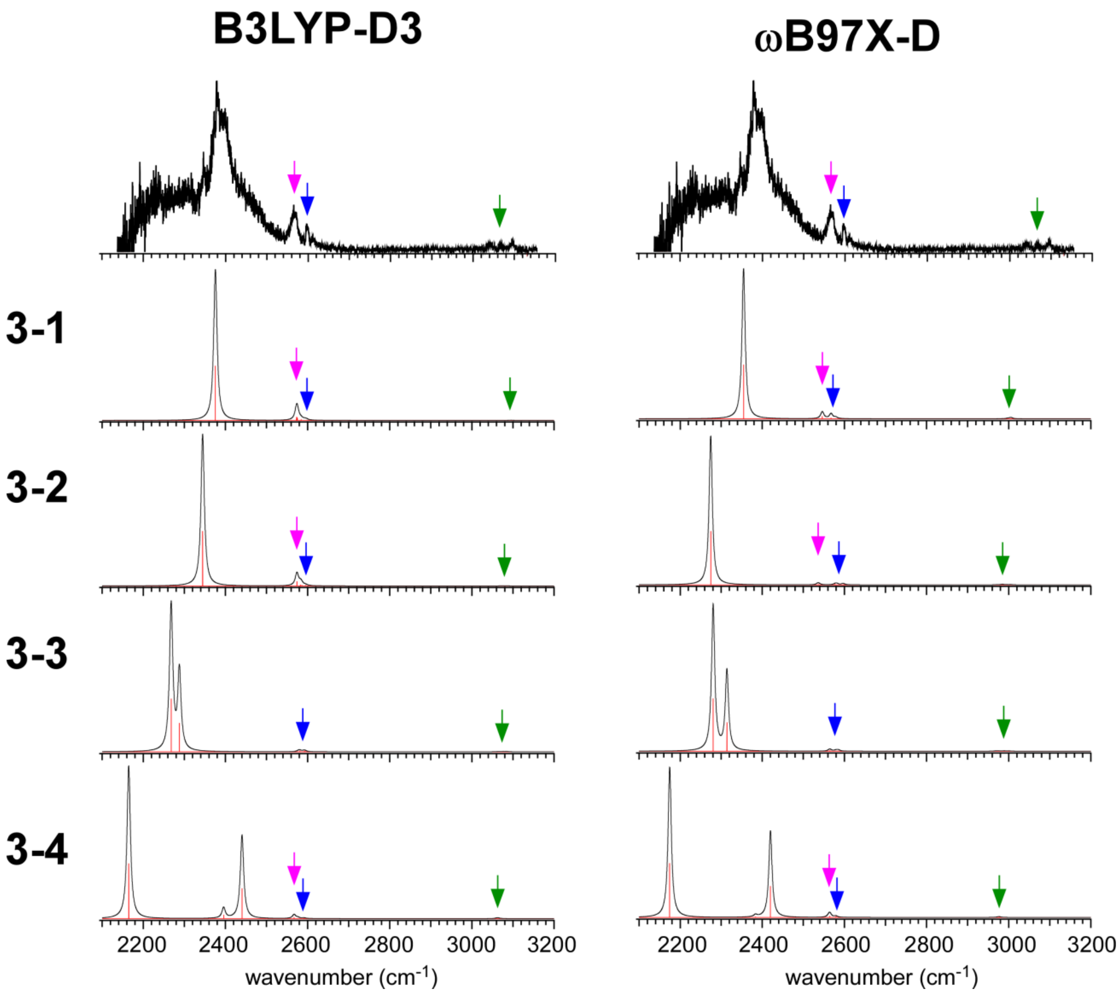

Figure S8. Comparison among the observed spectrum of $[Bz\text{-}(H_2S)_3]^+$ and simulated spectra. The simulated spectra were calculated at (left) B3-LYP-D3/6-311G+(3df,2p) and (right) ωB97X-D3/6-311G+(3df,2p) based on the energy-optimized structures shown in Figure S7(a) in ESI and Figure 7(a) in the main text, respectively. The same scaling factors as those in Figure 2 in the main text were applied. The colored arrows present the assignments of the bands; violet: free SH stretch of the ion core, blue: free SH stretch of the neutral H_2S moiety, and green: CH stretch. Each arrow indicates a peak of an envelope in which contribution of multiple vibrational modes can be involved. Bands without an arrow are attributed to H-bonded SH stretches of the ion core, and they appear only below 2450cm^{-1}. Note that no free SH stretch of the ion core is expected in the simulated spectra of **3-3** while such a band is clearly seen in the observed spectrum. The intensity in the observed band below 2200 cm^{-1} may have larger error than that in the higher frequency region because of the very weak IR light intensity.

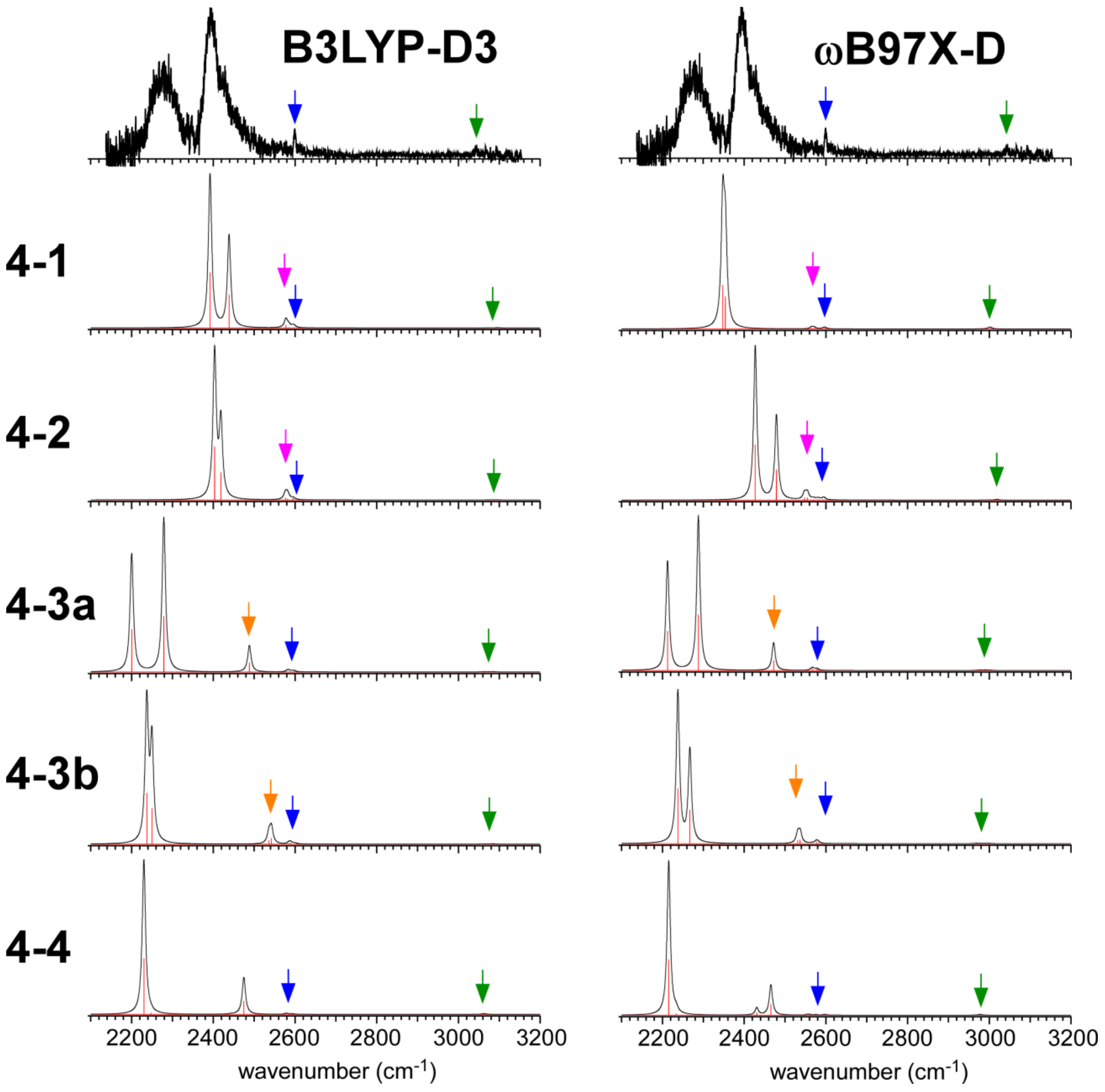

Figure S9. Comparison among the observed spectrum of $[Bz\text{-}(H_2S)_4]^+$ and simulated spectra. The simulated spectra were calculated at (left) B3-LYP-D3/6-311G+(3df,2p) and (right) ωB97X-D3/6-311G+(3df,2p) based on the energy-optimized structures shown in Figure S7(b) in ESI and Figure 7(b) in the main text, respectively. The same scaling factors as those in Figure 2 in the main text were applied. The colored arrows present the assignments of the bands; violet: free SH stretch of the ion core, blue: free SH stretch of the neutral H_2S moiety, orange: H-bonded SH stretch in the neutral moiety, green: CH stretch. Each arrow indicates a peak of an envelope in which contribution of multiple vibrational modes can be involved. Bands without arrow are attributed to H-bonded SH stretches of the ion core, and they appear only below 2500 cm^{-1}. Note that no free SH stretch of the ion core is expected in the simulated spectra of **4-4** and such a band is missing in the observed spectrum. The band intensity is the observed

band below 2200 cm^{-1} may have larger error than that in the higher frequency region because of the very weak IR light intensity.

Table S1. Relative energies of the stable structures of $[Bz\text{-}(H_2S)_2]^+$ calculated by four different functionals with the aug-cc-pVDZ basis set. In the calculations by each functional, the energy of the most stable isomer is set to zero. The zero point energy (ZPE) correction is applied. All units are kJ/mol/

	**2-1**	**2-2**	**2-3**	**2-4**
B3LYP-D3	0.0	6.8	4.0	10.6
M06-2X	7.6	17.7	5.5	0.0
M06-L	0.0	3.8	12.5	3.0
ωB97X-D	4.4	15.8	0.0	3.1
